# Human brain organoids containing microglia that have arisen innately adapt to a β-amyloid challenge better than those in which microglia are integrated by co-culture

**DOI:** 10.1186/s13287-024-03876-0

**Published:** 2024-08-13

**Authors:** Tyler J. Wenzel, Joseph D. Desjarlais, Darrell D. Mousseau

**Affiliations:** Cell Signalling Laboratory, Department of Psychiatry, College of Medicine, University of Saskatchewan, 107 Wiggins Road, Saskatoon, SK S7N 5E5 Canada

**Keywords:** Aβ, Alzheimer’s disease, Autopsy brain, Human brain, Microglial activation, Alzheimer disease, IBA1, Ionized calcium-binding adapter molecule 1, Neuroinflammation

## Abstract

**Background:**

Alzheimer disease (AD) is a heterogenous and multifactorial disease, and its pathology is partly driven by microglia and their activated phenotype. Brain organoids (BOs) are gaining prominence as a relevant model of the human brain for the study of AD; however, BOs are commonly devoid of microglia. To overcome this limitation, current protocols incorporate microglia through either (1) co-culture (BO co-culture), or (2) molecular manipulation at critical windows of BO development to have microglia arise innately (BO innate cultures). It is currently unclear whether the microglia incorporated into BOs by either of these two protocols differ in function.

**Methods:**

At in vitro day 90, BO innate cultures and BO-co-cultures were challenged with the AD-related β-amyloid peptide (Aβ) for up to 72 h. After Aβ challenge, BOs were collected for immunoblotting. Immunoblots compared immunodensity and protein banding of Aβ and ionized calcium-binding adapter molecule 1 (IBA1, a marker of microglial activation) in BOs. The translational potential of these observations was supported using 56 human cortical samples from neurocognitively normal donors and patients with early-onset AD and late-onset AD. Statistical analyses were conducted using the Kruskal–Wallis test, a two-way ANOVA, or a simple linear regression, and where applicable, followed by Dunn’s or Sidak’s test.

**Results:**

We show that BO co-cultures promote Aβ oligomerization as early as 24 h and this coincides with a significant increase in IBA1 levels. In contrast, the Aβs do not oligomerize in BO innate cultures and the IBA1 response was modest and only emerged after 48 h. In human cortical samples, we found IBA1 levels correlated with age at onset, age at death, and the putative diagnostic Aβ(1–42)/Aβ(1–40) ratio (particularly in their oligomeric forms) in a sex-dependent manner.

**Conclusions:**

Our unique observations suggest that BOs with innate microglia model the response of a healthy brain to Aβ, and by extension the initial stages of Aβ challenge. It would be impossible to model these early stages of pathogenesis in BOs where microglia are already compromised, such as those with microglia incorporated by co-culture.

**Supplementary Information:**

The online version contains supplementary material available at 10.1186/s13287-024-03876-0.

## Introduction

Brain organoids (BOs) are gaining prominence as a relevant model of the human brain for the study of neurodegenerative disorders, such as Alzheimer disease (AD). BO is an umbrella term for many different three-dimensional cultures, including a variety of unguided and regionalized models with their own advantages and limitations [1, 2]. Commonly used BO protocols are devoid of microglia, which are the innate immune cells of the brain [2]. This lack of microglia in BOs may represent a limitation in studies using BOs to examine AD-related pathology, given that microglial dysfunction is thought to contribute to AD pathology and play a significant role in β-amyloid peptide (Aβ) accumulation and deposition as dense-core plaques [3, 4]. To overcome this limitation, many studies resort to co-culturing induced pluripotent stem cell (iPSC)-derived microglia with BOs (BO co-cultures), as these cells will infiltrate the BO [5, 6]. However, the phenotype of microglia and their functions are known to irreversibly change when cultured outside a brain-like milieu [7]. As iPSC-derived microglia are commonly generated outside a brain-like milieu, it is unclear to what extent this altered function is retained when these cells are introduced into BOs by co-culture and, if retained, whether this altered function could unwittingly drive a phenotype and bias the interpretation of outcomes. An alternative method to incorporating microglia-like cells into BOs is by manipulating culture conditions of BOs so that these immune cells arise innately during the development of the BO and retain a more homeostatic, *i.e.* healthy, signature [8–10]. The latter approach will be referred to as BO innate cultures herein. To date there have not been any studies that have directly compared these cultures to determine which one may exhibit more relevant pathophysiological responses, but it is hypothesized that BO innate cultures may exhibit more translationally-relevant responses [11].

There are several accepted markers of microglia, including IBA1 (ionized calcium-adapter molecule 1), which is a cytoplasmic protein expressed by cells of a myeloid lineage and is thought to be involved in membrane ruffling, motility, and phagocytosis [12–14]. Systematic reviews conclude IBA1 does not increase in the AD brain across the entire patient population [12]. Although, in response to select stimuli, the expression of IBA1 increases in vitro and in vivo, and this increase is thought to reflect an ‘activated’ state as it is implicated in the increased phagocytic activity of microglia [15–17]. The AD-associated Aβs have been found to elicit a strong inflammatory response through the activation of microglia. For example, oligomeric and fibrillar Aβ induce different microglial phenotypes [18], and fibrillar Aβ burden precedes the activation of microglia in older mice that carry the APPSwe and PSEN1(ΔEx9) transgenes that are used to exacerbate AD-related amyloidosis [19]. Interestingly, in older (> 80 years) AD patients, the removal of amyloid plaques using anti-Aβ immunotherapy results in an increase in IBA1 expression, although this does not correlate with either total Aβ burden or Aβ(1–42) levels [20]. In general, there is a lack of direct evidence of the effects of different Aβs on the expression levels of IBA1 [3, 4, 12].

We chose to characterize IBA1 expression in BO co-cultures and BO innate cultures exposed to exogenous Aβs of different lengths, some of which have been shown to be neuroprotective, while others have been associated with AD-related pathology [21]. The translational potential of our observations was explored by comparing the IBA1 response in these experiments to IBA1 expression levels in 56 human cortical samples from neurocognitively normal donors as well as patients with early-onset AD (EOAD, onset < 65 years of age) and late-onset AD (LOAD, onset > 65 years of age). We demonstrate that BO co-cultures as well as BO innate cultures can take up the exogenous Aβs; however, BO co-cultures accumulate Aβ in the form of increasingly higher molecular weight species (e.g., oligomers), whereas Aβs in the BO innate cultures are detected primarily as monomers. The oligomeric Aβ species in BO co-cultures coincide with an increase in IBA1 levels, whereas the IBA1 response was milder and delayed in the BO innate cultures. These observations suggest that the co-cultured microglia may be functionally impaired, whereas the innate microglia have a capacity to manage an increase in Aβ availability. Thus, the BO innate cultures may give more translatable insight into how the ‘healthy’ brain could respond to increases in Aβs, and could, in turn, be exploited as a way to model some of earliest stages of amyloidosis associated with AD when the brain still has fully functional microglia-based adaptive mechanisms. Lastly, to clear up the conflicting reports implicating IBA1 changes in AD [12], we show the levels of IBA1 correlate with important disease characteristics, such as age at onset (AAO), age at death (AAD), the insoluble Aβ(1–42)/Aβ(1–40) ratio, and the soluble Aβ(1–42)/Aβ(1–40) ratio in a sex-dependent manner. Importantly, these correlations, with the exception of the soluble Aβ(1–42)/Aβ(1–40) ratio, are associated with samples obtained from donors diagnosed with EOAD. This suggests that targeting microglia for therapeutic intervention may provide less benefit in cases of AD that are diagnosed far later in life.

## Materials and methods

### Human tissues, antibodies, and reagents

Human autopsy cortical brain samples correspond to a mix of superior and middle frontal cortices (Brodmann Areas 9/46, respectively). These areas are associated with executive function and cognition [22] and show significant changes in, for example, transcription already at the earliest stages of disease [23]. The brain samples represent individuals with no cognitive deficits, EOAD or LOAD, and the demographic characteristics of these donors are summarized in Table 1. De-identified human donor information is detailed in Supplementary Table 1.

**Table 1:** Demographic characteristics of donors

Summary statistics	
**Sex (n, %)**	
Male	25, 44.64%
Female	31, 55.36%
**AD status (n, %)**	
Neurocognitively normal	**25**, **44.6%**
Male	12, 21.4%
Female	13, 23.2%
Early-onset AD	**15**, **26.8%**
Male	6, 10.7%
Female	9, 16.0%
Late-onset AD	**16**, **28.6%**
Male	7, 12.5%
Female	9, 16.1%
**Age, years (mean ± SD)**	
All donors	**70.2 ± 12.9**
Male	73.1 ± 10.1
Female	67.8 ± 14.3
Neurocognitively normal	**69.3 ± 11.6**
Male	70.7 ± 9.4
Female	68.1 ± 13.1
Early-onset AD	**59.0 ± 7.6**
Male	65.0 ± 2.7
Female	55.0 ± 7.2
Late-onset AD	**82.1 ± 7.6**
Male	84.3 ± 3.6
Female	80.3 ± 9.2

L-ascorbic acid, ethylenediaminetetraacetic acid (EDTA), heparin, Lowry assay kit (Peterson’s modification), protease inhibitor cocktail and sodium selenite were purchased from Millipore Sigma (Oakville, ON, Canada). Transforming growth factor-β1 (TGF-β1; cat# 100-21) was purchased from Peprotech (Cranbury, NJ, USA). Recombinant human transferrin (cat# 777TRF029) was purchased from InVitria (Fort Collins, CO, USA). Radioimmunoprecipitation assay buffer (RIPA) and the ROCK inhibitor Y-27632 (cat# 13624S) were purchased from Cell Signaling Technologies (Whitby, ON, Canada). EB formation media was purchased from STEMCELL Technologies (Vancouver, BC, Canada). Synthetic Aβ(1–38) (cat#: H-2966), Aβ(1–40) (cat# H-1194), and Aβ(1–42) (cat# H-1368) were obtained from Bachem Americas Inc. (Torrance, CA, USA) and the amino acid composition was confirmed by mass spectrometry as we have previously done [21]. Antibodies and their suppliers are provided in Table 2. All other reagents were sourced from Fisher Scientific (Ottawa, ON, Canada).

**Table 2:** List of antibodies used

Primary antibodies			
Target (for Western blotting)	Catalogue number	Dilution	Amount of protein loaded for western blotting (μg)
Rabbit anti-β-actin	Cell Signaling Technology Cat# 4970	1:5000	15
Mouse anti-GAPDH	Cell Signaling Technology Cat# 97166	1:2000	15
Rabbit anti-IBA1	Abcam Cat# ab178846	1:1000	15
Rabbit anti-TUBB3	Millipore Sigma Cat# T2200	1:5000	15
Mouse anti-β-amyloid(1–16)	Biolegend Cat# 803003	1:1000	15

Target (for immunohistochemistry)	Catalogue number	Dilution
Mouse anti-TMEM119	Millipore Sigma Cat# AMAb91528	1:200
Rabbit anti-IBA1	Abcam Cat# ab178846	1:200
Rabbit anti-TUBB3	Millipore Sigma Cat# T2200	1:200

Secondary antibodies		
Target (for Western blotting)	Catalogue number	Dilution
IRDye^®^ 680RD anti-rabbit IgG	LI-COR biosciences Cat# 926-68071	1:20,000
IRDye^®^ 680RD anti-mouse IgG	LI-COR biosciences Cat# 926-68070	1:20,000
IRDye^®^ 800CW anti-rabbit IgG	LI-COR biosciences Cat# 926-32211	1:20,000
IRDye^®^ 800CW anti-mouse IgG	LI-COR biosciences Cat# 926-32210	1:20,000

Target (for immunohistochemistry)	Catalogue number	Dilution
Donkey anti-Rabbit IgG, Alexa Fluor™ 594	ThermoFisher Scientific Cat# A21207	1:2000
Donkey anti-Rabbit IgG, Alexa Fluor™ 488	ThermoFisher Scientific Cat# A32790	1:2000
Goat anti-Mouse IgG, Alexa Fluor™ 488	ThermoFisher Scientific Cat# A11029	1:2000
Goat anti-Mouse IgG, Alexa Fluor™ 594	ThermoFisher Scientific Cat# A11005	1:2000

*TUBB3* βIII-tubulin, *IgG* immunoglobulin G, *IBA1* ionized calcium-binding adapter molecule 1, *GAPDH* glyceraldehyde 3-phosphate dehydrogenase, *TMEM119* transmembrane protein 119

### Inducible pluripotent stem cell (iPSC) maintenance

The UCSD087i-6-4 (87i, neurocognitively normal female donor) and UCSD086i-6-3 (86i, neurocognitively normal male donor) induced pluripotent stem cell sibling lines were purchased from WiCell (Madison, WI, USA). iPSCs were cultured feeder-free on 6-well plates coated with Matrigel™ human embryonic stem cell (hESC)-qualified matrix in a humidified 37 °C, 5% CO_2_ and 95% air atmosphere. iPSCs were maintained in iPSC basal media (Table 3) and supplemented with 1 ng/ml TGF-β1 and 25 ng/ml fibroblast growth factor 2 (FGF2).

**Table 3:** Induced pluripotent stem cell (iPSC) basal media used in all experiments

iPSC basal media
50:50 mixture of Dulbecco’s Modified Eagle Medium and Ham’s F-12 Nutrient Mixture (DMEM-F12)
15 mM N-2-hydroxyethylpiperazine-N-2-ethane sulfonic acid (HEPES)
2 mM L-alanyl-l-glutamine dipeptide
20 μg/ml recombinant human insulin
20 μg/ml recombinant human transferrin
20 ng/ml sodium selenite
0.2 mg/ml L-ascorbic acid

### Generation of unguided brain organoids (*BOs*) and iPSC-derived microglia

The protocol for generating human unguided BOs is described elsewhere [24, 25]. To create BO innate cultures, iPSCs were incubated for five min in 0.5 mM EDTA, removed from the plate, and resuspended at 9 × 10^4^ iPSCs/ml in iPSC basal media (Table 3) with 10 μM Y-27632 inhibitor and 25 ng/ml FGF2. 9 × 10^3^ iPSCs were seeded in a 96-well ultra-low attachment round-bottom plate. 24 h later, 100 μl of iPSC basal media were added to each well. On day five, embryoid bodies (EBs) were transferred to a 24-well ultra-low attachment plate (one EB per well) containing 500 μl of iPSC basal media and 1 μg/ml heparin. On day seven, media was replaced with 500 μl of ice-cold iPSC basal media containing 1X B27 without vitamin A and 3% v/v Matrigel, and on day 10, 500 μl of iPSC basal media containing 1X B27 with vitamin A was added to each well and incubated for seven days. Media was replaced weekly with 1250 µl of iPSC basal media containing 1X B27 with vitamin A. BOs were maintained at 37 °C in humidified 5% CO_2_ and 95% air atmosphere on an orbital plate shaker set at 0.118 g. BOs were harvested at day 90 for immunoblotting.

BO co-cultures were generated in a similar manner with minor modifications to prevent the arising of microglial populations innately. Specifically, the differences are that iPSCs were seeded in EB formation media (STEMCELL Technologies) with 10 μM Y-27632 inhibitor when generating BOs, and iPSC-derived microglia (from the same donor) were integrated by co-culture at day 60. iPSC-derived microglia were generated as described elsewhere [24, 26] with minor modifications. In brief, iPSCs were removed from the plate with 0.5 mM EDTA and transferred to a Matrigel-coated 12-well plate in iPSC basal media with 5 ng/ml bone morphogenetic protein 4 (BMP4), 25 ng/ml Activin A, 25 ng/ml FGF2 and 1 μM CHIR99021 (Media A). On day 2, half the media was removed and replaced with fresh Media A. On day 3, media was fully replaced with iPSC basal media with 50 ng/ml vascular endothelial growth factor (VEGF), 50 ng/ml stem cell factor (SCF), 25 ng/ml FGF2 and 10 μM SB431542. On day 5, media was replaced with iPSC basal media containing 50 ng/ml VEGF, 10 ng/ml SCF, 50 ng/ml interleukin (IL)-6, 10 ng/ml IL-3, 25 ng/ml FGF2, and 50 ng/ml thrombopoietin (Media B). Half-media changes with Media B were done until day 13. Non-adherent microglial precursor cells were transferred to a well of a Matrigel-coated 6-well plate at a density of 2.2 × 10^4^/cm^2^ in iPSC basal media with 100 ng/ml IL-34, 1X B27 with Vitamin A, and 25 ng/ml macrophage-colony stimulating factor (M-CSF). Until day 24, half-media changes were conducted every second day with iPSC basal media with 10 ng/ml IL-34, 1X B27 with Vitamin A, and 2.5 ng/ml M-CSF. On day 24, both adherent and non-adherent cells were transferred to a new Matrigel-coated 6-well plate at a density of 1 × 10^5^ cells/cm^2^ in iPSC basal media with 10 ng/ml IL-34, 2.5 ng/ml M-CSF, 50 ng/ml TGF-β1, 25 ng/ml cluster of differentiation 200 (CD200), 100 ng/ml fractalkine and 1X B27 with Vitamin A (microglia maintenance media). Half-media changes were conducted every second day with microglia maintenance media until day 34. On day 34, 5 × 10^5^ microglia-like cells were transferred to the 60-day in vitro BO cultures as described in other publications [5]. We have previously shown this differentiation protocol results in microglia that are TMEM119+ and IBA1+ [24].

### Treatment of BOs with β-amyloid peptides (Aβ)

Aβ species were reconstituted in hexafluoroisopropanol (HFIP) to disrupt any preexisting β-sheet structures [27]. HFIP was evaporated and Aβ species were reconstituted in sterile water prior to addition to BO cultures. Aβ species, including Aβ of 42 amino acids (Aβ(1–42)), Aβ of 40 amino acids (Aβ(1–40)), and Aβ of 38 amino acids (Aβ(1–38)), were used at 10 μM concentrations. At in vitro day 90, BOs were treated for 24–72 h with different Aβ species or its vehicle solution (water) and then were collected for immunoblotting or immunohistochemistry.

### Immunoblotting

BOs were homogenized in RIPA buffer containing protease inhibitor cocktail. Samples were triturated with a one-ml pipette, homogenized using a 22-gauge needle, and sonicated. Protein concentration was quantified by the Lowry assay and equalized to 0.5 μg/μl in 1% loading buffer (0.2 M Tris pH 6.8, 40% glycerol, 8% sodium dodecyl sulfate, 20% β-mercaptoethanol, 0.4% bromophenol blue). We avoided heating the samples so as to prevent protein aggregation.

For resolving Aβs, RIPA-soluble lysate fractions were resolved using a discontinuous 8 M urea/12% SDS-PAGE system as we have previously done for examining Aβs in human brain tissues and transferred onto a nitrocellulose membrane [21, 28]. As before, boiling the nitrocellulose membrane is critical for detection of the Aβs when using urea gel electrophoresis. Membranes were blocked in 1% bovine serum albumin (BSA) in TRIS-buffered saline (TBS: 25 mM Tris pH 7.4, 137 mM NaCl) and probed overnight (4 °C) with the 6E10 antibody (raised against residues 1–16 of the Aβ) diluted in 5% BSA in TBS-T (TBS with 0.1% Tween^®^20), washed thrice with TBS-T over 30 min, incubated with secondary fluorescently-labelled antibodies for one hour, and then washed again three times. For all other proteins, proteins were resolved on a 12% acrylamide gel, and resolved proteins were electroblotted onto a nitrocellulose membrane and blocked in 5% BSA in TBS for one hour. The duration of washes, incubation time with antibodies, and the solutions were identical to those used for Aβ immunoblotting. Details regarding protein loading and antibodies, including their catalogue numbers and dilutions, are summarized in Table 2. Proteins were visualized with a LI-COR Odyssey® Imager and densitometric analyses were done using the manufacturer’s software (Image Studio 5.3.5, LI-COR Biosciences, Lincoln, NE, USA).

### Immunohistochemistry

BOs were washed three times with sterile PBS and fixed in 4% paraformaldehyde for 16 h at 4 °C. 24 h later, the fixed organoids were incubated in a 15% sucrose-PBS solution for 24 h at 4 °C, followed by an additional 24 h in a 30% sucrose-PBS solution. Organoids were then embedded in optimal cutting temperature (OCT) compound in a mould. Tissues were flash frozen in a dry ice-ethanol bath, sectioned (15 μm) using a Leica CM1950 cryostat and microtome, and mounted on SuperFrost™ Plus microscope slides. Organoid sections were washed with PBS at room temperature for 5 min to remove OCT, immersed in blocking solution (5% normal donkey serum in a 0.1% Tween^®^20-PBS solution (PBS-T)) for one h, and incubated for 16 h at 4 °C in a humidified chamber with primary antibodies (Table 2) diluted in 5% normal donkey serum in PBS-T. After three washes with PBS-T over 30 min, organoid sections were incubated with secondary antibodies (Table 2) at room temperature in a humidified chamber for two h, followed by three additional washes with PBS-T. ProLong™ Glass Antifade Mountant with NucBlue™ was added and a coverslip placed on top. Slides were cured for 24 h at room temperature prior to imaging on a Zeiss AxioImager M.1 widefield microscope or an Olympus FV-1000 confocal microscope.

### Statistical analysis

Data (mean ± standard deviation (SD)) were analyzed using the (1) nonparametric Kruskal–Wallis test or Friedman’s test, followed by the Dunn’s or the Sidak’s post-hoc test, (2) the parametric two-way ANOVA, followed by Sidak’s post-hoc test, or (3) a simple linear regression (GraphPad Prism 9.2). Significance was established at *p* < 0.05. Where applicable, the Kruskal–Wallis H value, the Friedman’s value (Fr) and the two-way ANOVA F value are reported, which are used to calculate the *p* value. Higher H and Fr values indicate greater differences in medians, whereas higher F values indicate greater difference in means. Each human brain data-point was derived from a different donor (Supplementary Table 1), and the iPSC lines used to generate data-points are defined in captions. For BO immunoblotting experiments, five BOs from the same batch were collected in a microcentrifuge tube and homogenized. Five BOs yielded enough total protein per condition for immunoblotting experiments as we have previously described [29]. This was repeated across four (N = 4) independent batches of BOs grown on different days. Therefore, each BO data-point represents a different batch of organoid grown from the indicated iPSC line.

## Results

### Microglia are integrated into BOs using a co-culture or innate generation protocol

**Fig. 1:**
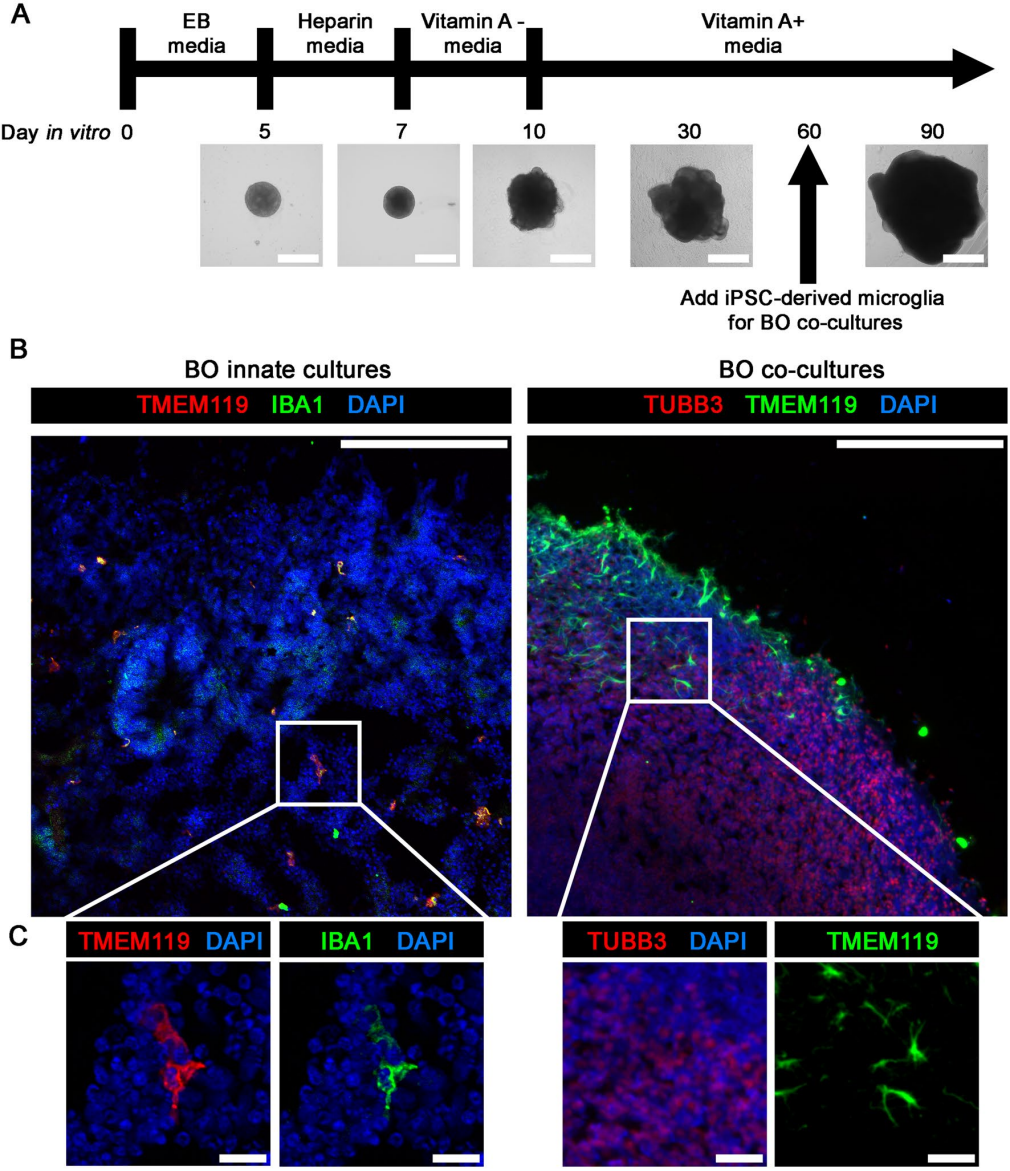
BOs were grown as described elsewhere [24, 25], and **A** BOs used in experiments exhibit the expected visual markers of proper development. Indirect immunofluorescence shows that (**B**, *left*) TMEM119+ and IBA1+ microglia cells are distributed throughout the tissue of BO innate cultures, whereas (**B**, *right*) BO co-cultures have areas of high microglia density and areas of no microglia. **C** High magnification inserts showing the morphology of microglia in each BO culture. Scale bars: **A** 1 mm, **B** 200 μm, and **C** 25 μm. All images were generated using BOs derived from the 87i iPSC line

Microglia, which are characterized by their co-expression of TMEM119 and IBA1 [30], were integrated into unguided BOs through either a co-culture or chemical method as we and others have previously described [5, 24, 25]. Figure 1A shows the schematic for generating BO innate cultures and BO co-cultures, as well as representative images of their development. BOs were cultured for 90 days, as we have recently shown this is a time point that BO innate cultures contain glia and neurons [25, 29]. At 90 days of culture, BO innate cultures expressed TMEM119+ and IBA+ microglia-like cells (Fig. 1B, *left*), and the TMEM119+ microglia-like cells (which were previously shown to be TMEM119+ and IBA1+ [24]) were present in BO co-cultures (Fig. 1B, *right*). The microglia in BO innate cultures also appeared to be more uniformly distributed throughout the tissues (Fig. 1B, *left*), whereas BO co-cultures had areas of high microglial density surrounded by areas that had none (Fig. 1B, *right*). The morphology of these microglia in BO innate cultures and BO co-cultures was also distinct (Fig. 1C).

### Exogenous Aβs accumulate and oligomerize in BO co-cultures, but not in BO innate cultures

**Fig. 2:**
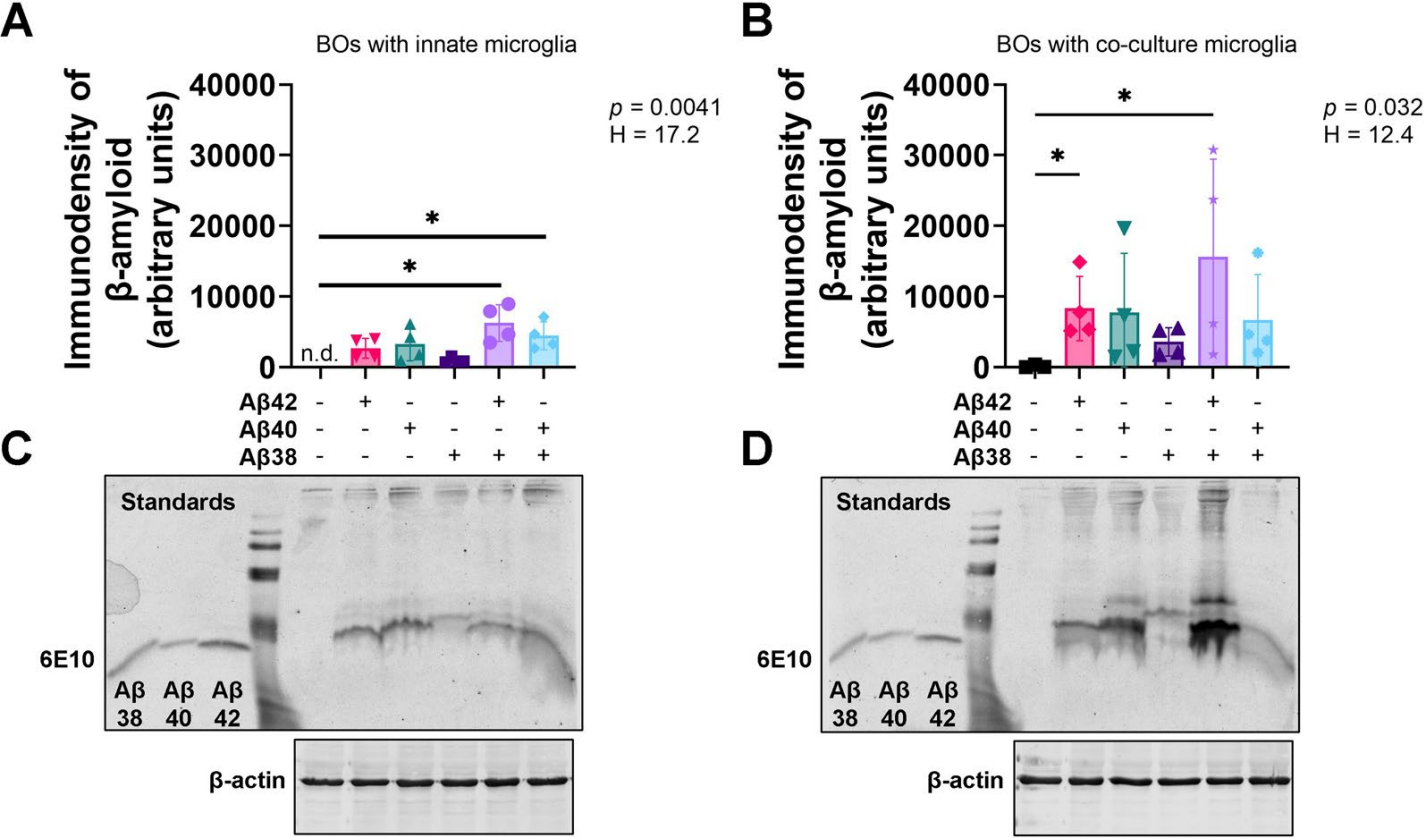
Aβs are processed more efficiently in **A** BOs with innate microglia than **B** BOs with co-culture microglia. 10 μM exogenous Aβs or its vehicle solution (water) was added to the culture media of BOs with **A** innate microglia or **B** co-culture microglia, and then cultures were incubated for 24 h. **A**, **B** Data from four independent experiments are presented as mean ± SD. *p < 0.05 according to the Dunn’s test following the randomized block Kruskal–Wallis test (*p* and H values shown on the figures). **C**, **D** Representative images of membranes probed with Aβ (6E10) or β-actin antibodies are shown, and were used in densitometric analyses to generate the graphs shown in **A**, **B**. **A–D** Figures were generated using the 87i iPSC line. Each data-point or lane was derived by pooling five organoids from a different batch of organoids (batches defined as BOs generated on different days from iPSCs of a different vial). **A** n.d. = not detected

We have previously demonstrated that Aβs added to culture medium can penetrate BOs, and we have shown that Aβ species oligomerize within 24 h in other systems [21, 24]. However, it is unclear how the presence of microglia can alter Aβ accumulation and fibril behaviour. We chose to test this using BOs, which are a translationally relevant model of the human brain. We treated BO innate cultures and BO co-cultures with exogenous Aβs of different lengths for 24 h. Immunoblotting revealed Aβs could be detected in the lysate of BO innate cultures (Fig. 2A), but at levels lower than those in BO co-cultures (Fig. 2B). Unlike the Aβs in BO innate cultures (Fig. 2C), the Aβs in the BO co-cultures were clearly oligomerized, with some combinations of Aβs, namely Aβ(1–42) and Aβ(1–38), inducing a strong ~ 10 kDa dimer (Fig. 2D). Levels of the housekeeping protein β-actin were used for normalizing data as well as demonstrating equal loading across lanes (Fig. 2C, D).

### IBA1 expression differs between BO co-cultures and BO innate cultures in response to exogenous Aβs

**Fig. 3:**
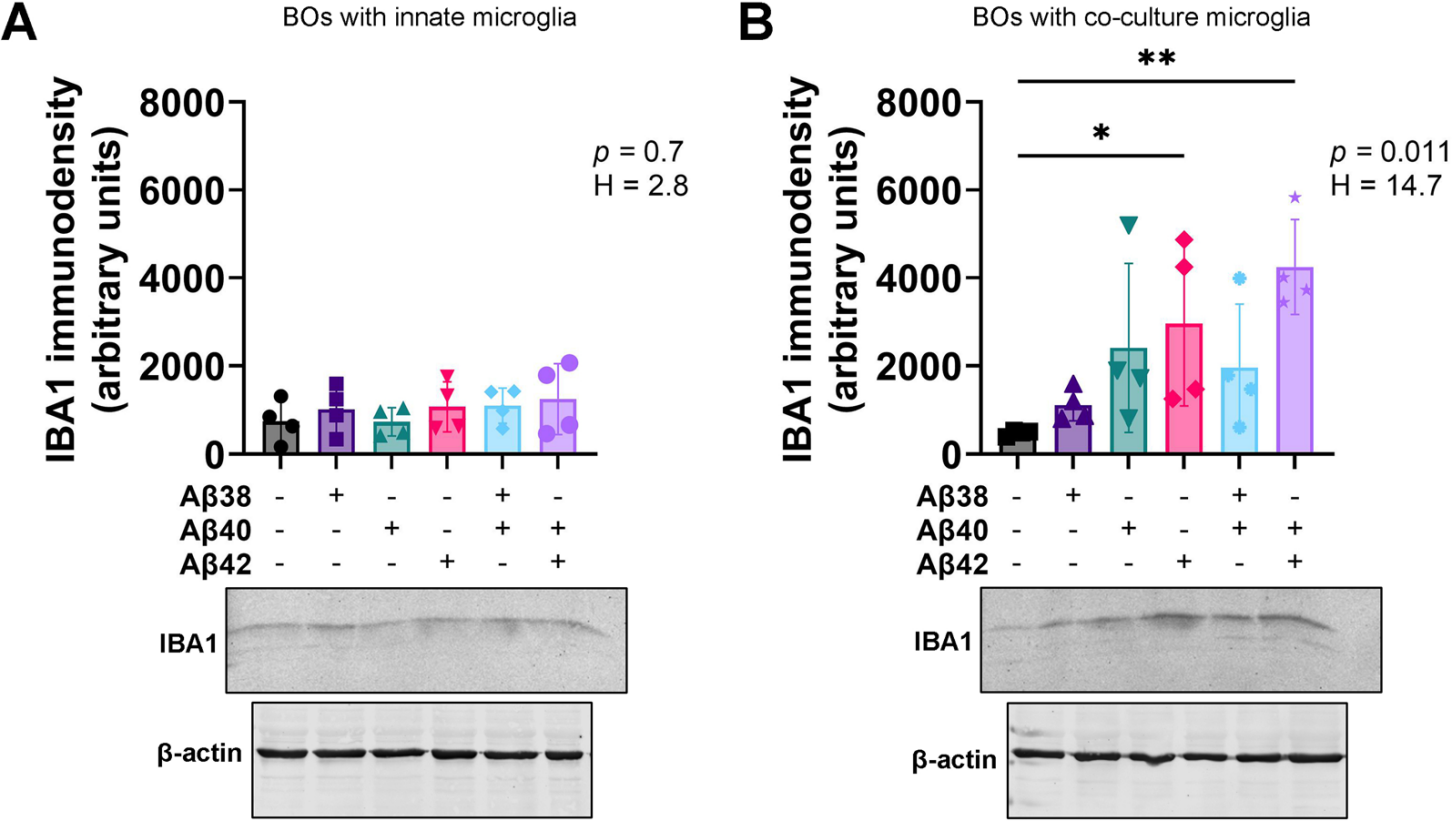
In response to the 24 h Aβ treatments shown in Fig. 1, IBA1 levels increase in **B** BOs with co-culture microglia but not **A** BOs with innate microglia. **A**, **B** Data from four independent experiments are presented as mean ± SD. *p < 0.05, **p < 0.05 according to the Dunn’s test following the randomized block Kruskal–Wallis test (*p* and H values shown on the figures). **A**, **B** Representative images of membranes probed for IBA1 and β-actin are shown, and were used in densitometric analyses to generate the graphs shown. Figures were generated using the 87i iPSC line. Each data-point or lane was derived by pooling five organoids from a different batch of organoids (batches defined as BOs generated on different days from iPSCs of a different vial)

**Fig. 4:**
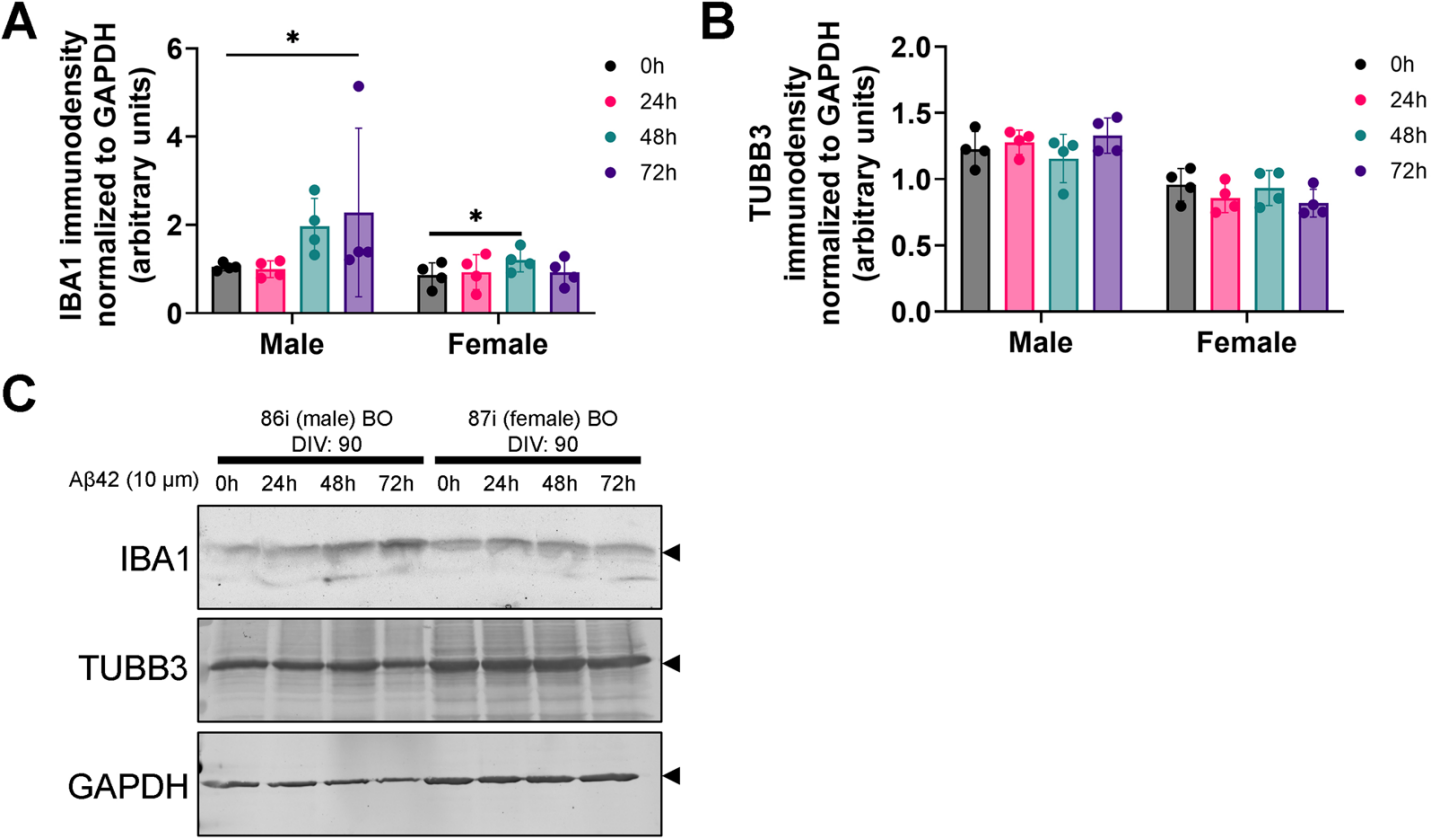
BO innate cultures were treated with Aβ(1–42) for up to 72 h. **A** IBA1 levels increase in male BOs by 72 h of Aβ(1–42)-exposure and female BOs showing an increase by 48 h of Aβ(1–42)-exposure. **B** TUBB3 levels did not change in either male or female BOs over the 72-h Aβ(1–42) treatment. **A**, **B** Data from four independent experiments are presented as mean ± SD. *p < 0.05 according to the Dunn’s test following a Friedman’s test (**A** Male: *p* < 0.01, Fr = 9.6; Female: *p* = 0.051, Fr = 7.5; **B** Male: *p* = 0.51, Fr = 2.7; Female: *p* = 0.32, Fr = 3.9). **C** Representative images of membranes probed for IBA1, TUBB3 and GAPDH are shown, and were used in densitometric analyses to generate the graphs shown in **A**, **B**. Proteins of interest are demarcated with arrows. **A–C** Figures generated using the 86i (male) and 87i (female) iPSC lines. Each data-point or lane was derived by pooling five organoids from a different batch of organoids (batches defined as BOs generated on different days from iPSCs of a different vial)

The literature suggests Aβs activate human microglia in a near IBA1-independent manner [3, 4, 12]. We measured the expression of IBA1 in our BO co-culture and BO innate culture lysates. The IBA1 levels of BO innate cultures did not increase after a 24 h exposure to any of the Aβs **(**Fig. 3A**)**, whereas the IBA1 levels of BO co-cultures increased after exposure to Aβ(1–42) and a combination of Aβ(1–42) plus Aβ(1–40) **(**Fig. 3B**)**. Representative immunoblots are shown below the graphs. We then investigated whether BO innate cultures could respond to a prolonged exposure to Aβ(1–42). The IBA1 levels of BO innate cultures increased after being exposed to Aβ(1–42) for 48 h and 72 h in female and male BOs, respectively **(**Fig. 4A**)**, but densitometric analyses indicated that these IBA1 levels were still lower than those observed in BO co-cultures exposed to Aβ(1–42) for only 24 h (Fig. 3B). As Aβ(1–42) exposure is neurotoxic [21, 31], possibly through a neuroinflammatory mechanism [32, 33], we tested whether this Aβ(1–42) exposure in BO innate cultures resulted in a loss of the neuronal protein TUBB3 (β3-Tubulin). TUBB3 levels in BO innate cultures were not affected by Aβ(1–42) exposures at any time-point tested (Fig. 4B). Representative immunoblots, including those of GAPDH (glyceraldehyde 3-phosphate dehydrogenase), are shown in Fig. 4C.

### Autopsy brain tissue reveals a correlation between IBA1 expression in the female Alzheimer disease (AD) brain and age at onset (AAO), but not duration of the disease itself

A variety of factors have been considered for their possible influence on IBA1 levels in the context of AD, including *APOE* and *TREM2* risk variants, dementia, AD histopathology, and AD Braak staging [14, 34–38]. In contrast, there are reports that do not support any change in the number of IBA1+ cells in the AD brain [39–42]. A systematic review on the topic concluded that IBA1 levels do not categorically increase or decrease across all AD patients [12]. This is not unexpected as AD is a multifactorial and heterogenous disease, with several types and classes of this neurodegenerative disorder—some of which implicate an inflammatory phenotype, where others do not—being reported using different analytical modalities [43–45]. Pooling of data across broad categories of patients with a heterogeneous disease limits our understanding of AD, whereas identifying trends within patient populations may help to inform on treatments that may work for a specific subset of patients and in turn support consideration of a personalized medicine approach to management of AD.

Aside from two studies considering risk variants [14, 37], prior studies investigating the relationship of IBA1 and AD predominately categorized patient samples based on a broad clinical diagnosis of AD and did not consider individual patient demographics, such as AAO (defined herein as the age at first clinical presentation of symptoms), AAD, symptom duration, the Aβ(1–42)/Aβ(1–40) ratio, which is used as a putative diagnostic of disease [46–48], or sex, which has been implicated as a risk factor for certain types of AD [28, 49]. Therefore, we set out to identify whether IBA1 immunodensity is altered in cortical samples across a range of patient demographics, which are summarized in Table 1 and detailed in Supplementary Table 1.

**Fig. 5:**
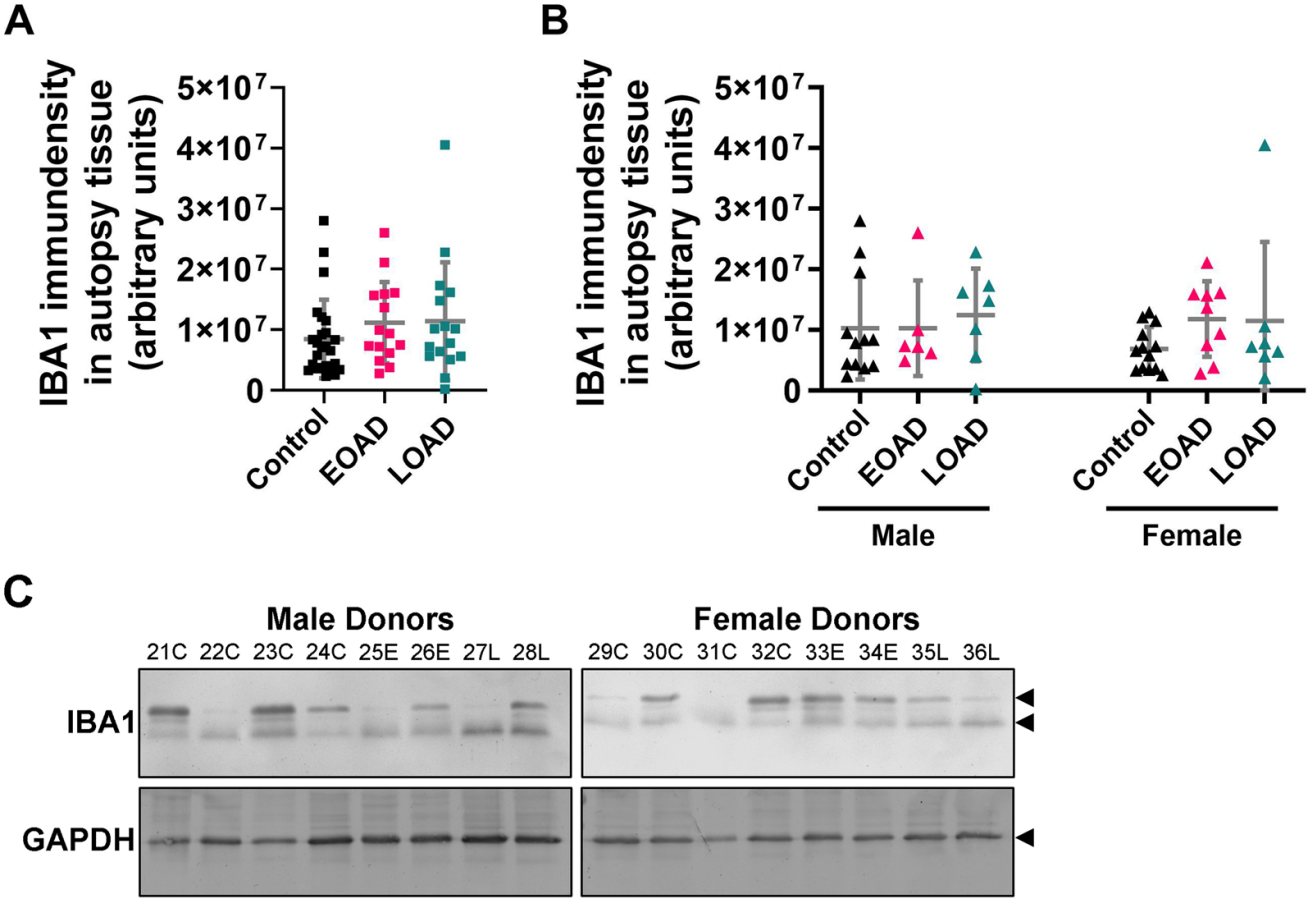
IBA1 levels do not change in autopsy tissue in an AD-state dependent manner. **A** IBA1 levels in autopsy brain tissue were the same in neurocognitively normal (control), EOAD, and LOAD donors. **B** Stratifying IBA1 levels by sex did not reveal sex dependent differences. Data from 56 donors are presented as mean ± SD. No significance (*p* > 0.05) **A** according to the Dunn’s test following the randomized block Kruskal–Wallis test (Krusak-Wallis: *p* = 0.49, H = 1.4) and **B** according to a Sidak’s post-hoc test following a randomized block two-way ANOVA (Two-way ANOVA: *p*(*sex*) = 0.67, F = 0.19; *p*(*disease state*) = 0.38, F = 0.98; *p*(*interaction*) = *0.63, F* = *0.46*). **C** Representative images of membranes probed for IBA1 and GAPDH are shown, and were used in densitometric analyses to generate the graphs shown in **A**, **B**. Proteins of interest are demarcated with arrows. Letter in column header, *e.g.,* ‘23C’ or ‘25E’, indicates the diagnosis (C = neurocognitively normal, E = EOAD, L = LOAD), while the number is the identifier (ID) for referencing the patient age, cause of death, and apolipoprotein E alleles on Supplementary Table 1. Data normalized to GAPDH are shown in Supplementary Fig. 1, and all immunoblots used to generate these graphs are presented in Supplementary Figs. 2, 3

Cortical IBA1 levels do not increase in either EOAD (< 65 years of AAO) or LOAD (> 65 years of AAO) samples (Fig. 5A), and no differences are detected when stratifying by sex (Fig. 5B). Representative immunoblots demonstrating the diversity of IBA1 band patterns between patients are shown (Fig. 5C). Normalizing IBA1 levels to the housekeeping protein GAPDH does not reveal any significant differences in cortical IBA1 levels between EOAD and LOAD patients and neurocognitively normal donors (Supplementary Fig. 1). To appreciate the heterogeneity of IBA1 levels, GAPDH and IBA1 immunoblots for all human samples are shown in Supplementary Figs. 2, 3.

**Fig. 6:**
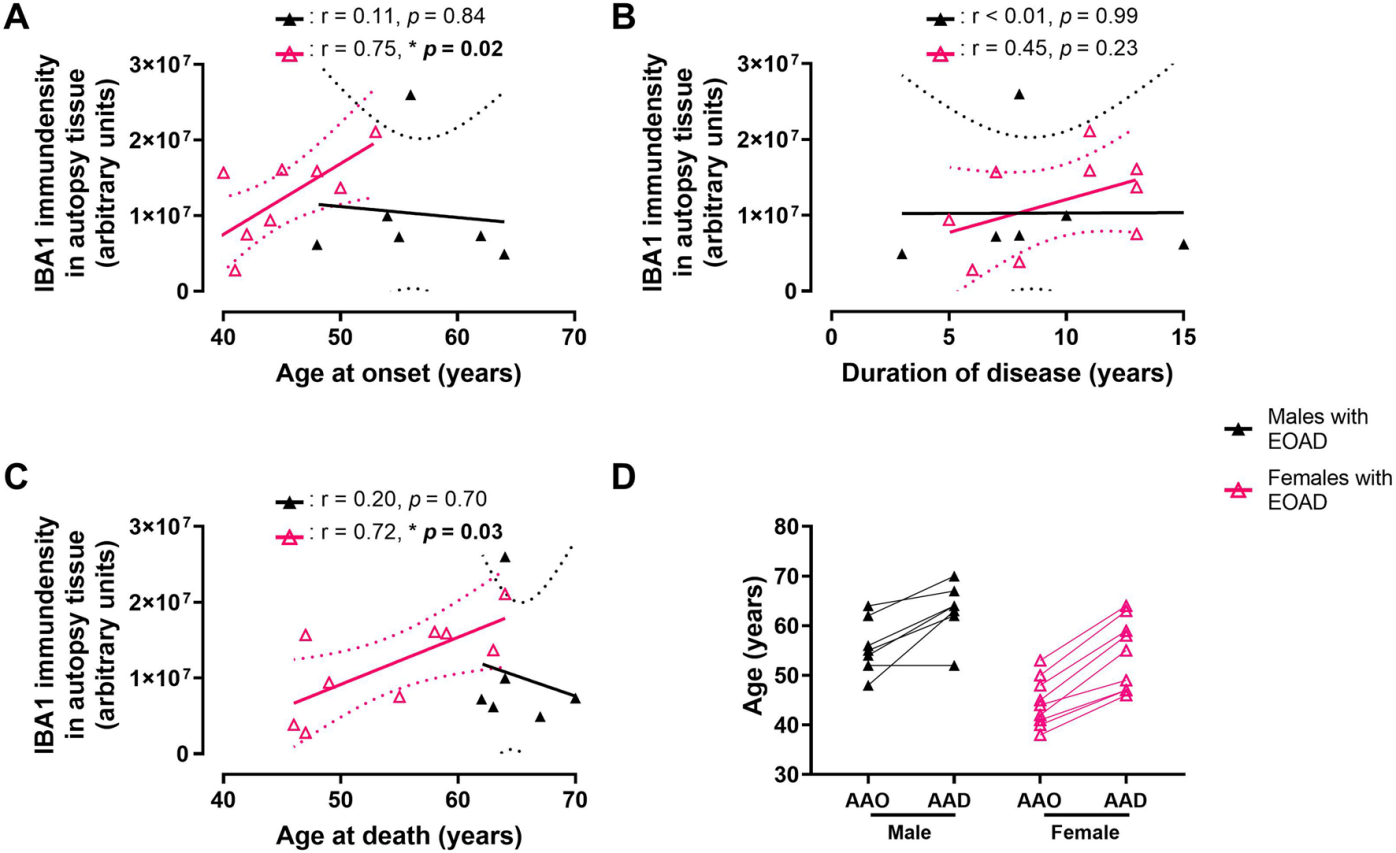
IBA1 levels in autopsy brain tissue from EOAD donors were stratified by sex and plotted against donor characteristics, including **A** age at onset (AAO), **B** duration of disease, and **C** age at death (AAD). IBA1 levels in autopsy tissue from female human donors with EOAD positively correlate with **A** age at onset (AAO) and **C** age at death (AAD), but **B** did not correlate with duration of disease. **A–C** IBA1 levels in autopsy tissue from male donors did not correlate with any donor characteristic tested. Correlation coefficients (r) and their corresponding *p* value (*p < 0.05) according to a simple linear regression are shown. **A–C** Solid line denotes the linear regression and the dashed lines represent 95% confidence intervals. **D** Solid line denotes the age at onset (which is indicative of the age at first clinical presentation of symptoms) and age at death for each EOAD donor. Legends for colors and symbols are shown on the right of the figure. There was no correlation between IBA1 levels and the tested patient characteristics in EOAD autopsy brain tissue when data was not stratified by sex, nor was there any correlation between these variables in LOAD samples (see Supplementary Table 2)

Next, we conducted linear regression analysis to identify any relationships between IBA1 immunodensity and demographic characteristics of human brain donors. Levels of IBA1 show a strong positive correlation with AAO and AAD in females with EOAD, but not males (Fig. 6A, C, Supplementary Table 2). IBA1 levels do not correlate with duration of symptoms, nor with AAO in individuals with LOAD regardless of sex (Fig. 6B, Supplementary Table 2). While the males and females with EOAD exhibit similar duration of disease (Fig. 6B), females are younger at first clinical presentation, i.e., AAO (Fig. 6D).

**Fig. 7:**
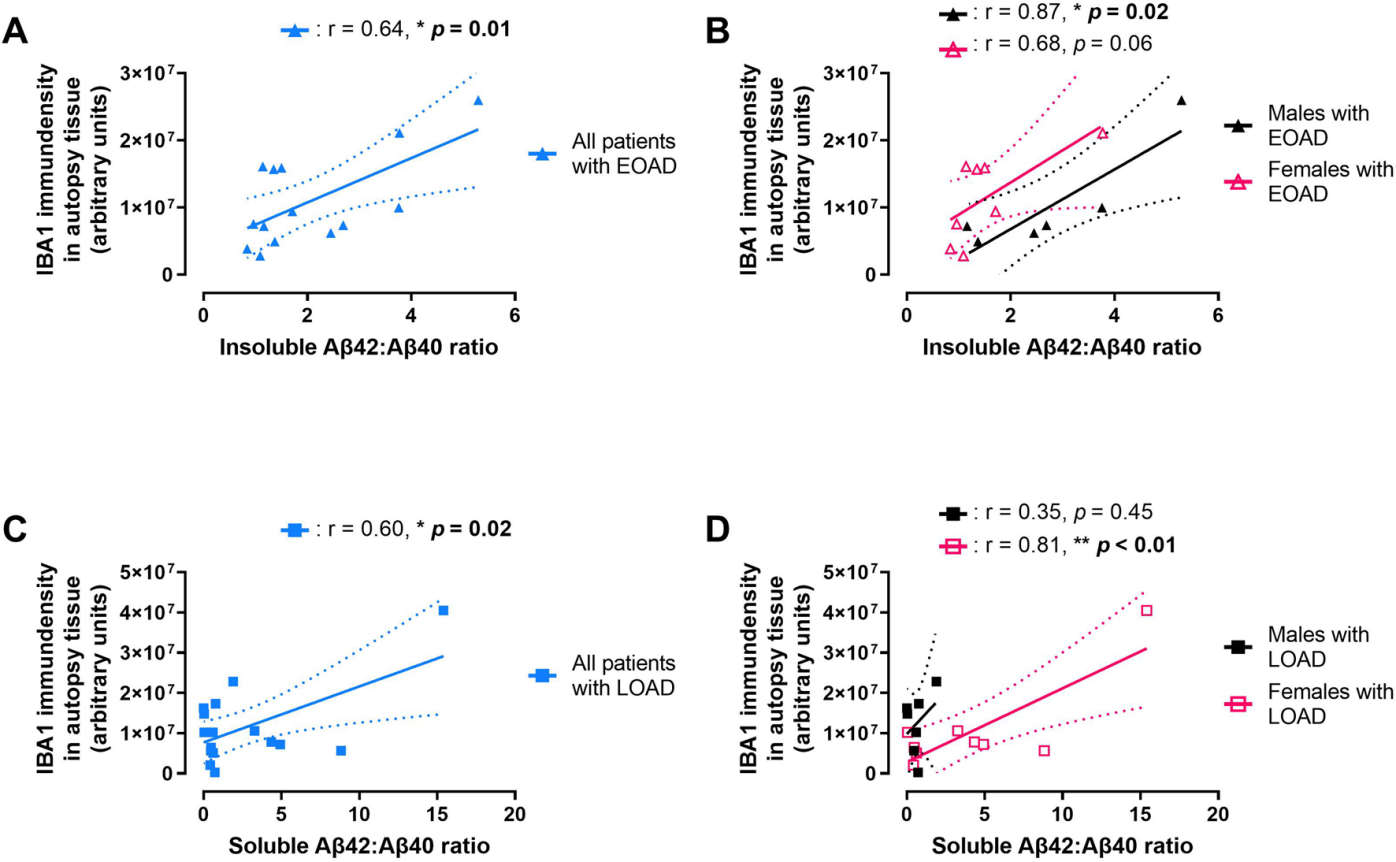
IBA1 levels in autopsy brain tissue from **A**, **B** EOAD donors plotted against the ratio of insoluble Aβ(1–42)/Aβ(1–40) in the same tissues or **C**, **D** LOAD plotted against the ratio of soluble Aβ(1–42)/Aβ(1–40). IBA1 levels in autopsy tissue positively correlate with **A** the ratio of insoluble Aβ(1–42)/Aβ(1–40) in EOAD patients, and **C** the ratio of soluble Aβ(1–42)/Aβ(1–40) in LOAD patients. When stratified by sex, **B** there was a positive correlation between IBA1 levels and the insoluble Aβ(1–42)/Aβ(1–40) ratio in males with EOAD, but only a positive trend with females with EOAD. **D** In LOAD, IBA1 levels and the soluble Aβ(1–42)/Aβ(1–40) ratio was positively correlated in males, but not females. Correlation coefficients (r) and their corresponding *p* value (*p < 0.05, **p < 0.01) according to a simple linear regression are shown. **A–C** Solid line denotes the linear regression and the dashed lines represent 95% confidence intervals. **D** Solid line denotes the age at onset and age at death for each EOAD donor. Legends for colors and symbols are shown on the right of each panel. As summarized in Supplementary Table 3, 4, there was no correlation between IBA1 levels and the soluble Aβ(1–42)/Aβ(1–40) ratio in EOAD patients, nor was there a correlation between IBA1 levels and insoluble Aβ(1–42)/Aβ(1–40) ratio in LOAD patients

Given IBA1 levels are implicated in microglial phagocytosis, which plays a central role for removing Aβs from the brain [12–14, 50], we examined whether IBA1 immunodensity correlated with levels of the insoluble (plaque-associate) and soluble forms of Aβs in our AD brain samples. For these analyses, we used the levels of Aβs in these same donors that were measured in our recent publications [21, 28], and representative blots of these densitometric analyses are shown in Supplementary Fig. 4. IBA1 immunodensity showed a moderate positive correlation with insoluble Aβ(1–42)/Αβ(1–40) ratio in patients with EOAD (Fig. 7A), and this correlation was stronger in male samples than female samples (Fig. 7B). IBA1 immunodensity also had a moderate positive correlation with the soluble Aβ(1–42)/Aβ(1–40) ratio in patients with LOAD (Fig. 7C), and stratifying by sex reveals this correlation was strongly associated with female patients, but not male patients (Fig. 7D). Supplementary Table 3 shows the insoluble Aβ(1–42)/Aβ(1–40) ratio had no correlation with IBA1 immunodensity in samples from LOAD patients, and Supplementary Table 4 reveals the soluble Aβ(1–42)/Aβ(1–40) ratio had no correlation in samples from EOAD patients. Supplementary Tables 3, 4 also show the relationship between IBA1 immunodensity and individual Aβ species. A preliminary investigation of phosphoTau(Ser396) levels in these samples did not reveal any association with levels of IBA1 (*data not shown*).

## Discussion

BOs have been used to study a range of different diseases, from pediatric white matter diseases such as leukodystrophies to adult neurodegenerative diseases such as AD [6, 51–53]. However, BOs are commonly devoid of the microglia which exhibit key functions that can mitigate or drive disease and associated symptoms [6, 50, 54–57]. Therefore, the lack of microglia in BO models may unintentionally enhance pathology and bias any conclusions drawn from the resulting data. This could ultimately interfere with the translation of any data derived from these microglia-null BOs to the human disease context [6, 54]. In fact, it has been demonstrated that incorporating microglia in BOs dramatically changes their neural network activity and their secretory profile [58, 59]. There are currently two commonly used methods for generating BOs with microglia-like cells: BO innate cultures and BO co-cultures [5, 8, 25], and we have previously shown that BO innate cultures express proteins at levels and molecular weights similar to the human brain parenchyma [25]. It is important to note that there exist studies claiming to use microglia-containing BOs, but in several of these reports the cultures are spheroids and not BOs as defined by the recent nomenclature consensus; these misnomers may complicate any interpretation of the literature that refers to any number of different culture platforms as ‘BOs’ [1, 60]. To the best of our knowledge, despite there existing a hypothesis that BO co-cultures have dysfunctional microglia [2, 11], there have not been any studies that have: (1) investigated whether the microglia-like cells in these BOs are dysfunctional and exhibit different responses to immunostimulants, such as Aβ; (2) validated which types of microglia-containing BOs display a more pathophysiologically relevant response; nor (3) demonstrated which type of BOs are more representative of a brain in a healthy or disease state.

Our data demonstrate differential responses of BO innate cultures and BO co-cultures to exogenous Aβs, indicating that the method of integrating microglia into BOs could impact microglia-dependent phenotypes. Interestingly, unlike Fagerlund et al. [61], who reported no detectable change in IBA1 levels in response to immunostimulants, we detected IBA1 changes in BO co-cultures treated with Aβ. However, we note that Fagerlund et al. [61] incorporate iPSC-derived microglia precursor cells and let them differentiate in the BOs in a self-directed manner, whereas we incorporate terminally differentiated iPSC-derived microglia. Furthermore, as previously suggested, any future studies would need to test multiple timepoints of introducing microglia-like cells, as this could influence cell maturation and functionality and affect experimental parameters [2, 59]. Regardless, we highlight the protocol used to incorporate microglia into BOs has overt effects on the immune phenotype of these cultures. It is clear from our data that these models are different, and future studies need to take this critical difference into consideration.

There are many BO models that incorporate microglia-like cells, but our study is the only one that has directly compared cellular responses of the two most used methodologies to generate BOs with microglia. We examined changes in the IBA1 levels of BO innate cultures and BO co-cultures in response to exogenously administered Aβs, and compared these results to IBA1 levels in autopsy human brain tissue. We demonstrate for the first time that these models exhibit strikingly different responses to Aβs. For example, BO co-cultures facilitate the oligomerization of Aβs and, as mentioned above, exhibit a rapid (within 24 h) and substantial increase in IBA1 levels in response to these disease-associated peptides, unlike BO innate cultures which maintain Aβs in a monomeric form and showed a milder and delayed IBA1 response that emerges at 48 h. We hypothesize that the response of BO co-cultures were not physiological because their creation relies on a monoculture of microglia and it is known that the transcriptome of cultured microglia are irreversibly altered [7].

Our studies show that IBA1 levels are similar between AD patients with EOAD and LOAD, which corroborate previous studies [12]. However, we extend knowledge by showing there is a strong correlation between IBA1 levels and AAO or AAD, but only in females with EOAD. Furthermore, the levels of IBA1 correlate strongly with the Aβ(1–42)/Aβ(1–40) ratio, which has been shown to be representative of clinical AD progression [48] whether measured as insoluble (plaque-associated) or soluble [21, 28]. Interestingly, the ratio of the plaque-associated Aβs, which are a strong trigger for inflammation [62], only correlates with IBA1 levels in cases of EOAD, regardless of sex. In contrast, the soluble Aβs, which trigger a lesser inflammatory phenotype, but also exert synaptotoxic events [63], correlate with IBA1, but only in cases of LOAD; this correlation in LOAD cases is driven specifically by females. These observations suggest two things: first, the roles of IBA1-elevated microglia differ in their influence on Aβ behaviour between cases of EOAD (accelerated progression) and LOAD (protracted progression); second, the role of IBA1-elevated microglia may differ significantly between the male and female AD brain. This may explain the conflicting reports between IBA1 levels and a diagnosis of disease if cases of AD are indiscriminately pooled, as is often the standard practice [12]. Thus, the microglia in BO co-cultures are impaired and may be a better model of EOAD, whereas our BO innate cultures treated with Aβs may be a more representative model of the more common later onset forms of AD, when the brain may still have a proportionally larger pool of functional microglia. Any models of AD in the literature are, for the most part, representative of EOAD and aggressive amyloidosis. Our BO innate culture model provides a promising, translationally relevant human model of the human late-onset AD brain. This could facilitate marker development and drug development for this intractable disease.

## Conclusions

Due to the heterogeneous nature of AD [43–45], there is a growing call for developing personalized treatment plans for patients, as it is clear one treatment may not benefit all patients equally [64–67]. This, unfortunately, may require re-analyzing past data, or repeating past studies with more robust data collection. For example, most studies we identified that investigated the relationship of IBA1 and AD had relied on pooling samples into broad patient categories, such as those with a clinical diagnosis of any type of AD and those that are neurocognitively normal, and thus did not overtly consider any subtypes of AD or patient demographics [14, 34–42]. Similarly, our recent report [10] summarizing articles using AD BOs further highlights the lack of consideration in disease heterogeneity or risk factors in many studies modelling AD. For example, out of the 18 AD BO studies we discussed, none considered sex as a risk factor. We also noted many of these studies used enzyme-linked immunosorbent assays (ELISAs) for Aβ quantification which are not equally sensitive in differentiating Aβ species, nor in distinguishing mono- and oligomeric states of the peptides. Our data herein underscore the importance of robust data collection, as there would have been no correlation in our study between IBA1 levels and AD donors if samples from LOAD and EOAD patients had been pooled, nor would there have been correlations if our study was not relatively sex balanced. The same lack of significant result may be extrapolated to drug development as, for example, patients with certain demographic characteristics may benefit more from a particular treatment. To create effective therapies for AD, it is likely that models that better represent the human disease are needed, as well as improved diagnostic tools to identify subtypes of AD. Our findings suggest that BO innate cultures may be a model more representative of a healthy brain’s response to Aβ insults, whereas the microglia in BO co-cultures are impaired and may be a better model of microglial dysfunction in neurodegenerative diseases.

### Supplementary Information

Supplementary Material 1.

## Data Availability

All data generated or analysed during this study are included in this published article its supplementary information files, with the exception of the β-amyloid peptide and phosphorylated Tau data from the donor brains used in this study. These data were published in another article and are available from DDM on reasonable request.

## Abbreviations

Aβ: β-Amyloid peptide
Aβ(1–38): Aβ of 38 amino acids
Aβ(1–40): Aβ of 40 amino acids
Aβ(1–42): Aβ of 42 amino acids
AAD: Age at death
AAO: Age at onset
AD: Alzheimer disease
APOE: Apolipoprotein E
BO: Brain organoid
BO co-culture: BOs with iPSC-derived microglia incorporated by co-culture
BO innate culture: BOs with microglia-like cells arising innately due to specific molecular cocktails
BSA: Bovine serum albumin
CD200: Cluster of differentiation 200
DMEM-F12: 50:50 Mixture of Dulbecco’s Modified Eagle Medium and Ham’s F-12 Nutrient Mixture
EB: Embryoid body
EDTA: Ethylenediaminetetraacetic acid
ELISA: Enzyme-linked immunosorbent assay
EOAD: Early-onset Alzheimer disease
FGF2: Fibroblast growth factor 2
hESC: Human embryonic stem cell
HEPES: N-2-hydroxyethylpiperazine-N-2-ethane sulfonic acid
HFIP: Hexafluoroisopropanol
IBA1: Ionized calcium-binding adapter molecule 1
IgG: Immunoglobulin G
IL: Interleukin
iPSC: Induced pluripotent stem cell
GAPDH: Glyceraldehyde 3-phosphate dehydrogenase
LOAD: Late-onset Alzheimer disease
M-CSF: Macrophage-colony stimulating factor
n.d.: Not detected
phosphoTau: Phosphorylated tau
RIPA: Radioimmunoprecipitation assay buffer
SD: Standard deviation
SCF: Stem cell factor
TBS: TRIS-buffered saline
TBS-T: TBS with 0.1% Tween^®^20
TGF-β1: Transforming growth factor-β1
TUBB3: β3-Tubulin
VEGF: Vascular endothelial growth factor
WB: Western blotting
