# Supplementary material for: Human brain organoids containing microglia that have arisen innately adapt to a β-amyloid challenge better than those in which microglia are integrated by co-culture
Source: Stem Cell Res Ther. 2024 Aug 13;15:258. doi: 10.1186/s13287-024-03876-0 (PMC11320858; doi:10.1186/s13287-024-03876-0)
Supplement: Supplementary file 1 — Supplementary Material 1. [file 13287_2024_3876_MOESM1_ESM.docx]

# Supplementary Figures and Their Legends


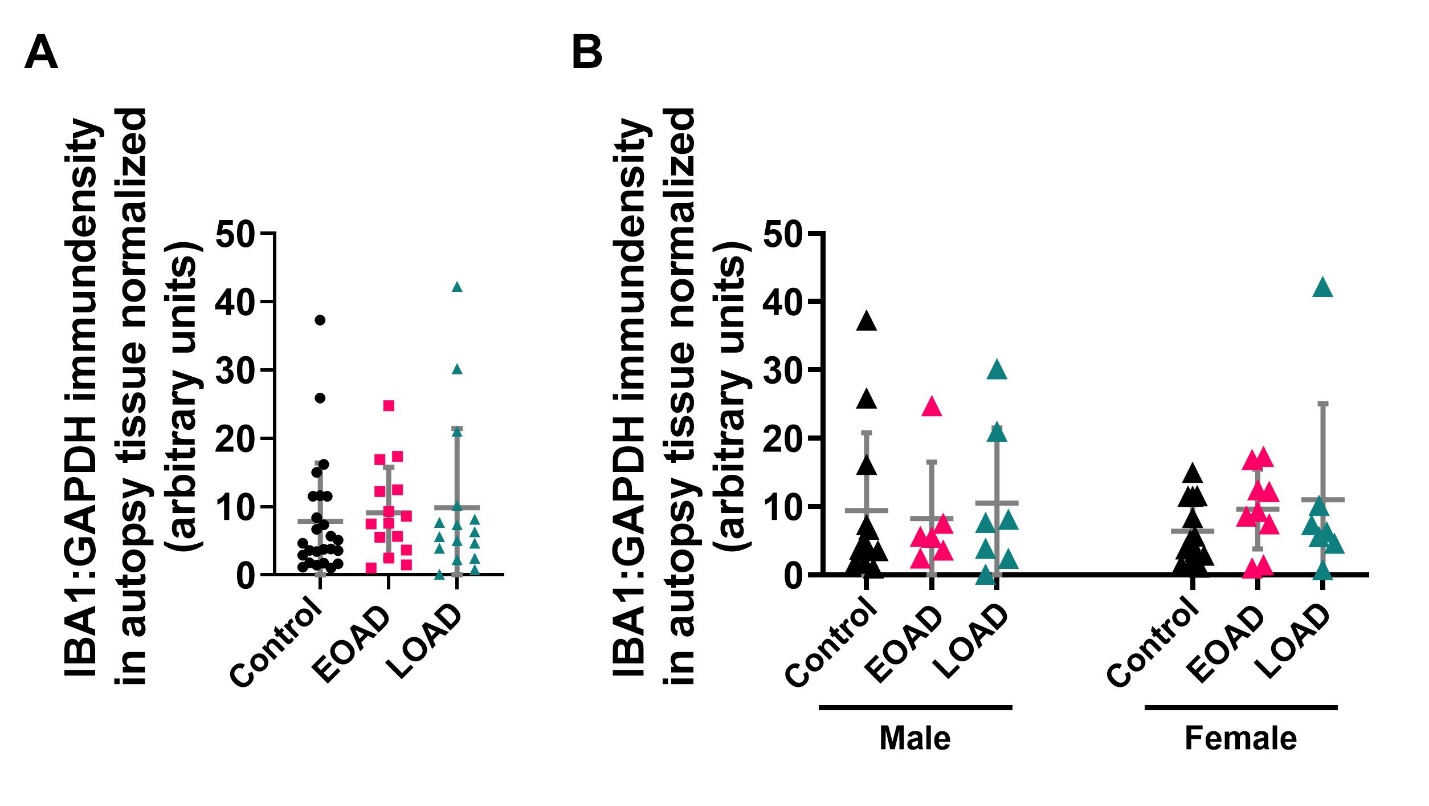
**Supplementary Figure 1:** IBA1 levels normalized to GAPDH do not change in autopsy tissue in an AD-state dependent manner. **(A)** IBA1 levels in autopsy brain tissue were the same in neurocognitively normal (control), EOAD, and LOAD donors. **(B)** Stratifying IBA1 levels by sex did not reveal sex dependent differences. Data from 56 donors are presented as mean ± SD. No significance (*p* > 0.05) **(A)** according to the Dunn’s test following the randomized block Kruskal-Wallis test (Krusak-Wallis: *p* = 0.49, H = 1.4) and **(B)** according to a Sidak’s post-hoc test following a randomized block two-way ANOVA (Two-way ANOVA: *p(sex)* = 0.67, F = 0.19; *p(disease state)* = 0.38, F = 0.98; *p(interaction) = 0.63, F = 0.46*). **(C)** Representative images of membranes probed with the IBA1 and GAPDH antibodies to generate graphs are shown in **Figure 5**, and were used in densitometric analyses to generate the graphs shown in **(A,B)**. All immunoblots used to generate these graphs are presented in **Supplementary Figures 2,3**.


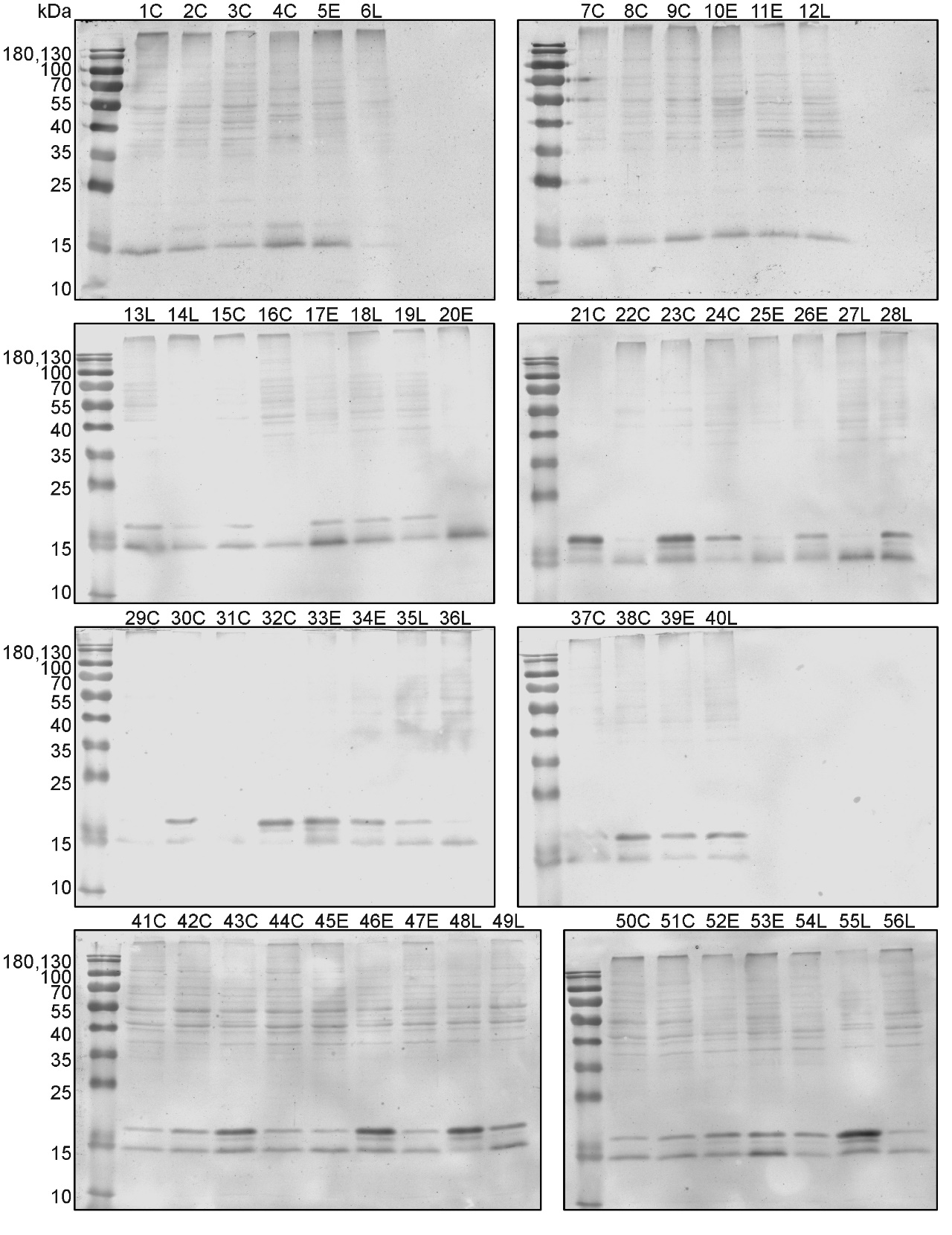


**Supplementary Figure 2:** Uncropped immunoblots probing for IBA1 in human cortical tissues. Letter on column header indicates disease status (C = neurocognitively normal, E = EOAD, L = LOAD) and number is the identifier (ID) for referencing the patient age, cause of death, and apolipoprotein E alleles on **Supplementary Table 1**.


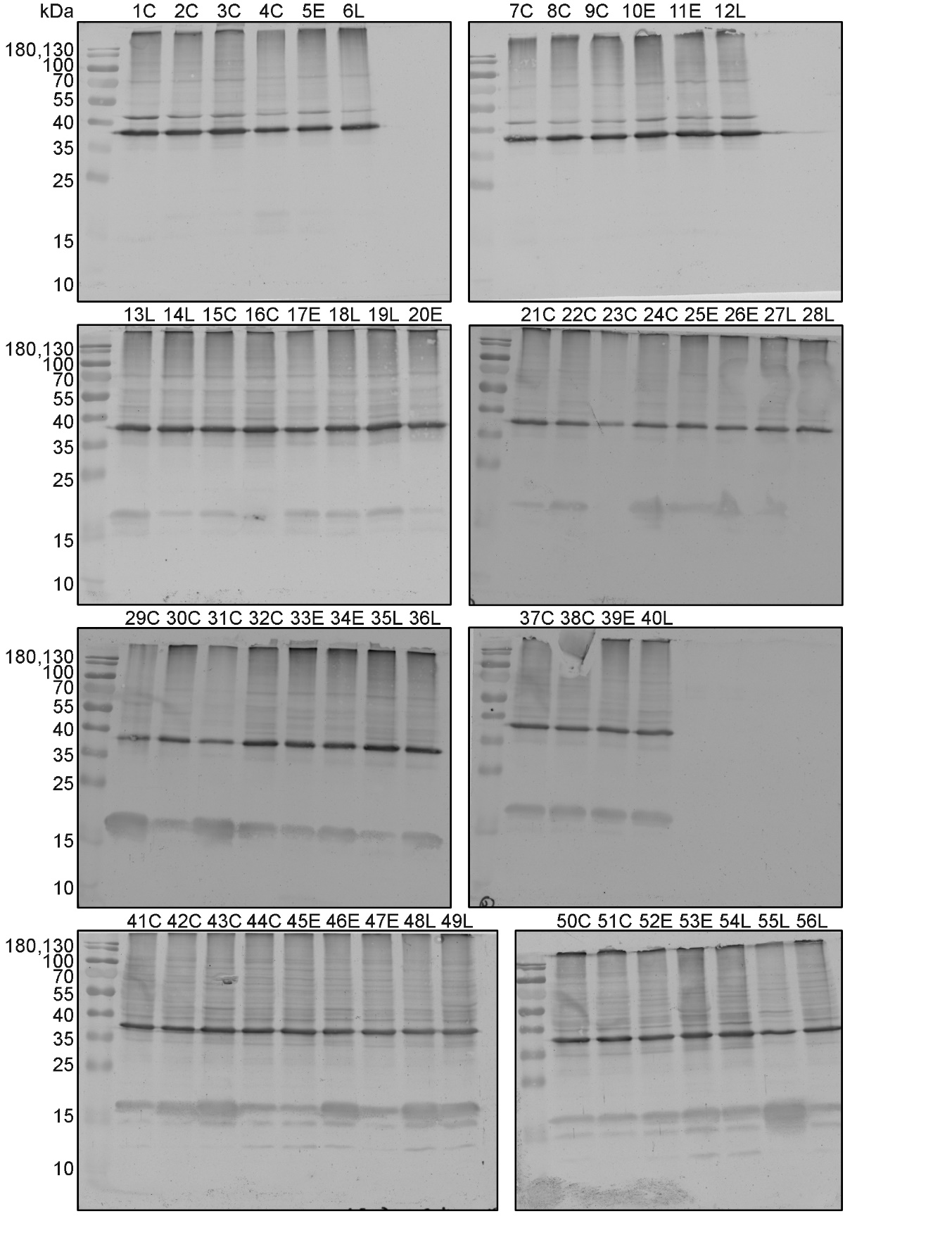


**Supplementary Figure 3:** Uncropped immunoblots probing for GAPDH in human cortical tissues. Letter on column header indicates disease status (C = neurocognitively normal, E = EOAD, L = LOAD) and number is the identifier (ID) for referencing the patient age, cause of death, and apolipoprotein E alleles on **Supplementary Table 1**.


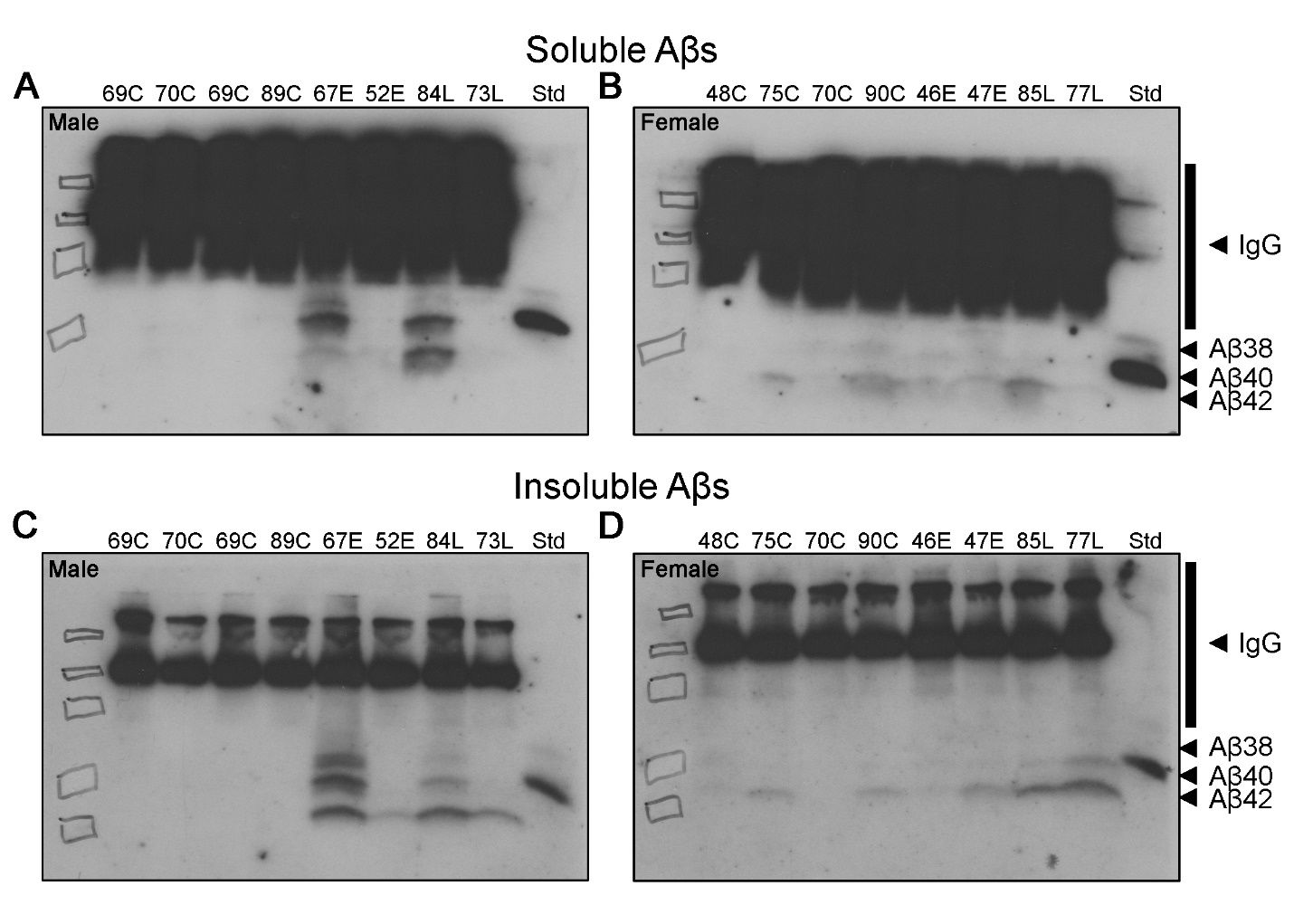


**Supplementary Figure 4:** Blots representing **(A, B)** soluble (aqueous) and the corresponding **(C, D)** insoluble (plaque-associated) Aβs. Samples are separated by samples from **(A,C)** male and **(B,D)** female donors. 20-30 mg wet weight brain tissue was homogenized in 400-600 μl ice-cold RIPA buffer and then centrifuged at 12,000 *g* for 10 min at 4°C. The RIPA-soluble fraction contains the soluble Aβs. The pellet collected after centrifugation was dissolved in 5 M guanidine hydrochloride for 2 h at room temperature, and the resulting solution contains the insoluble Aβs. Protein concentration was quantified by the Lowry assay, and 300 μg of the RIPA-soluble and guanidine-soluble fraction was used for the immunoprecipitation (which occurred over 16 h at 4°C) of Aβs using 6E10 anti-Aβ antibodies. 6E10 antibodies target all Aβs with an intact N-terminal sequences, *i.e.,* contain residues 1-17 of the Aβ sequence. The antibody-Aβ complexes were precipitated using protein A/G Sepharose, and the individual immunocomplexes were resolved on a discontinuous 8 M urea gel system [21,28]. This system allows for resolving Aβ length variants, which were detected by blotting with 6E10 antibodies and visualized using enhanced chemiluminescence. Densitometric analyses were performed using ImageJ 1.32j. Letter on column header indicates disease status (C = neurocognitively normal, E = EOAD, L = LOAD) and number is the patient age. *Note: Aβ(1-40) standard = Std; Immunoglobulin heavy and light chains of 6E10 antibodies used to immunoprecipitated Aβs = IgG.*

# Supplementary Tables

**Supplementary Table 1:** Sex, age, apolipoprotein E status, and cause of death for each human brain sample used in this study.

| **Alzheimer Disease (AD) Status** | **ID** | **Sex** | **Age** | **Apolipoprotein E alleles** | **Cause of death** |
| --- | --- | --- | --- | --- | --- |
| Neurocognitively normal (C) | 1 | Male | 69 | ε3/ε4 | Heart failure |
|  | 2 | Male | 70 | ε3/ε4 | Hemorrhagic colitis |
|  | 3 | Male | 69 | ε2/ε4 | Heart failure |
|  | 4 | Male | 89 | ε3/ε3 | Heart failure |
|  | 21 | Male | 79 | ε3/ε3 | Acute pulmonary edema |
|  | 22 | Male | 85 | ε3/ε3 | Heart failure |
|  | 23 | Male | 60 | ε2/ε3 | No discernable cause |
|  | 24 | Male | 61 | ε3/ε3 | Pancreatic metastasis |
|  | 41 | Male | 59 | ε3/ε3 | Not known |
|  | 42 | Male | 64 | ε4/ε3 | Not known |
|  | 43 | Male | 77 | ε3/ε3 | Not known |
|  | 44 | Male | 66 | ε3/ε3 | Not known |
|  | 7 | Female | 48 | ε3/ε4 | Breast cancer metastasis |
|  | 8 | Female | 75 | ε3/ε3 | Coronary arteriosclerosis |
|  | 9 | Female | 70 | ε3/ε3 | Retroperitoneal hematoma |
|  | 15 | Female | 75 | ε3/ε3 | Pulmonary edema |
|  | 16 | Female | 77 | ε3/ε3 | Pancreatic carcinoma |
|  | 29 | Female | 76 | ε4/ε3 | Heart failure |
|  | 30 | Female | 86 | ε3/ε3 | Heart failure |
|  | 31 | Female | 50 | ε3/ε3 | Heart failure |
|  | 32 | Female | 51 | ε3/ε3 | Breast cancer |
|  | 37 | Female | 59 | ε3/ε3 | Chronic obstructive pulmonary disease |
|  | 38 | Female | 64 | ε3/ε3 | Cecal adenocarcinoma |
|  | 50 | Female | 91 | ε3/ε3 | Not known |
|  | 51 | Female | 63 | ε3/ε3 | Not known |
| Early-onset AD (EOAD) | 5 | Male | 67 | ε2/ε3 | Early-onset AD |
|  | 25 | Male | 63 | ε4/ε3 | Early-onset AD |
|  | 26 | Male | 64 | ε3/ε3 | Early-onset AD |
|  | 45 | Male | 62 | ε4/ε4 | Not known |
|  | 46 | Male | 64 | ε4/ε3 | Not known |
|  | 47 | Male | 70 | ε4/ε4 | Not known |
|  | 10 | Female | 46 | ε3/ε4 | Early-onset AD |
|  | 11 | Female | 47 | ε3/ε3 | Early-onset AD |
|  | 17 | Female | 58 | ε3/ε3 | Early-onset AD |
|  | 20 | Female | 59 | ε3/ε4 | Early-onset AD |
|  | 33 | Female | 47 | ε4/ε3 | Early-onset AD |
|  | 34 | Female | 49 | ε4/ε3 | Early-onset AD |
|  | 39 | Female | 62 | ε4/ε3 | Early-onset AD |
|  | 52 | Female | 63 | ε4/ε3 | Not known |
|  | 53 | Female | 64 | ε3/ε3 | Not known |
| Late-onset AD (LOAD) | 6 | Male | 84 | ε2/ε3 | Late-onset AD |
|  | 13 | Male | 88 | ε3/ε4 | Late-onset AD |
|  | 14 | Male | 88 | ε3/ε4 | Late-onset AD |
|  | 27 | Male | 79 | ε4/ε3 | Late-onset AD |
|  | 28 | Male | 85 | ε4/ε3 | Late-onset AD |
|  | 48 | Male | 79 | ε4/ε4 | Not known |
|  | 49 | Male | 87 | ε4/ε3 | Not known |
|  | 12 | Female | 77 | ε3/ε4 | Late-onset AD |
|  | 18 | Female | 74 | ε3/ε4 | Late-onset AD |
|  | 19 | Female | 85 | ε3/ε3 | Late-onset AD |
|  | 35 | Female | 76 | ε4/ε4 | Late-onset AD |
|  | 36 | Female | 80 | ε3/ε3 | Late-onset AD |
|  | 40 | Female | 61 | ε4/ε3 | Late-onset AD |
|  | 54 | Female | 87 | ε4/ε3 | Not known |
|  | 55 | Female | 90 | ε4/ε3 | Not known |
|  | 56 | Female | 93 | ε4/ε3 | Not known |

**Supplementary Table 2:** Relationship of cortical IBA1 levels and patient demographics according to a linear regression

| Cortex |  | Control |  | EOAD |  | LOAD |  |
| --- | --- | --- | --- | --- | --- | --- | --- |
| IBA1 vs AAO |  | N/A |  | *p* = 0.6515 |  | *p* = 0.6744 |  |
|  |  | N/A |  | r = 0.1272 |  | r = 0.1139 |  |
|  | by sex | Male | Female | Male | Female | Male | Female |
|  |  | N/A | N/A | *p* = 0.8414 | ***p* = 0.0211** | *p* = 0.4533 | *p* = 0.4115 |
|  |  | N/A | N/A | r = 0.1061 | **r = 0.7457** | r = 0.3416 | r = 0.3134 |
| IBA1 vs duration |  | N/A |  | *p* = 0.3804 |  | *p* = 0.7166 |  |
|  |  | N/A |  | r = 0.2442 |  | r = 0.1067 |  |
|  | by sex | Male | Female | Male | Female | Male | Female |
|  |  | N/A | N/A | *p* = 0.9928 | *p* = 0.2261 | *p* = 0.8390 | *p* = 0.8456 |
|  |  | N/A | N/A | r = 0.0048 | r = 0.4483 | r = 0.1077 | r = 0.0827 |
| IBA1 vs AAD |  | N/A |  | *p* = 0.4020 |  | *p* = 0.3683 |  |
|  |  | N/A |  | r = 0.2337 |  | r = 0.2411 |  |
|  | by sex | Male | Female | Male | Female | Male | Female |
|  |  | N/A | N/A | *p* = 0.7030 | ***p*** **= 0.0298** | *p* = 0.6109 | *p* = 0.3262 |
|  |  | N/A | N/A | r = 0.2007 | **r = 0.7167** | r = 0.2357 | r = 0.3705 |

*Note: age at death (in years) = AAD; age at onset (in years) = AAO; duration of disease (in years) = duration; early-onset Alzheimer disease = EOAD; late-onset Alzheimer disease = LOAD*

**Supplementary Table 3:** Relationship of cortical IBA1 levels and insoluble Aβs according to a linear regression

| Cortex | IBA1 vs Aβs  (insoluble) | Control |  | EOAD |  | LOAD |  |
| --- | --- | --- | --- | --- | --- | --- | --- |
| Aβ(1-40) |  | *p* = 0.1609 |  | ***p*** **= 0.0214** |  | *p* = 0.2200 |  |
|  |  | r = 0.2892 |  | **r = 0.5871** |  | r = 0.3245 |  |
|  | by sex | Male | Female | Male | Female | Male | Female |
|  |  | *p* = 0.3001 | *p* = 0.1994 | *p* = 0.2588 | *p* = 0.0541 | *p* = 0.3580 | *p* = 0.4643 |
|  |  | r = 0.3266 | r = 0.3807 | r = 0.5495 | r = 0.6580 | r = 0.4123 | r = 0.2808 |
| Aβ(1-42) |  | *p* = 0.2083 |  | ***p* = 0.0450** |  | *p* = 0.8913 |  |
|  |  | r = 0.2606 |  | **r = 0.5240** |  | r = 0.0372 |  |
|  | by sex | Male | Female | Male | Female | Male | Female |
|  |  | *p* = 0.4225 | *p* = 0.1791 | *p* = 0.2411 | *p* = 0.1878 | *p* = 0.0935 | *p* = 0.3006 |
|  |  | r = 0.2557 | r = 0.3971 | r = 0.5666 | r = 0.4830 | r = 0.6780 | r = 0.3892 |
| Aβ(1-42)/Aβ(1-40) ratio |  | *p* = 0.3097 |  | ***p* = 0.0131** |  | *p* = 0.4175 |  |
|  |  | r = 0.2117 |  | **r = 0.6430** |  | r = 0.2179 |  |
|  | by sex | Male | Female | Male | Female | Male | Female |
|  |  | *p* = 0.4196 | *p* = 0.3495 | ***p*** **= 0.0231** | *p* = 0.0612 | *p* = 0.5175 | *p* = 0.6036 |
|  |  | r = 0.2572 | r = 0.2806 | **r = 0.8732** | r = 0.6843 | r = 0.2972 | r = 0.2012 |

*Note: β-amyloid peptide = Aβ; early-onset Alzheimer disease = EOAD; late-onset Alzheimer disease = LOAD*

**Supplementary Table 4:** Relationship of cortical IBA1 levels and soluble Aβs according to a linear regression

| Cortex | IBA1 vs Aβs  (soluble) | Control |  | EOAD |  | LOAD |  |
| --- | --- | --- | --- | --- | --- | --- | --- |
| Aβ(1-38) |  | *p* = 0.3845 |  | *p* = 0.8901 |  | *p* = 0.1711 |  |
|  |  | r = 0.1881 |  | r = 0.0387 |  | r = 0.3597 |  |
|  | by sex | Male | Female | Male | Female | Male | Female |
|  |  | *p* = 0.8304 | *p* = 0.1081 | *p* = 0.6084 | P = 0.5495 | ***p* = 0.0113** | *p* = 0.6909 |
|  |  | r = 0.0693 | r = 0.4665 | r = 0.2675 | r = 0.2312 | **r = 0.8682** | r = 0.1548 |
| Aβ(1-40) |  | *p* = 0.2803 |  | ***p* = 0.0272** |  | *p* = 0.4644 |  |
|  |  | r = 0.2247 |  | **r = 0.5681** |  | r = 0.1971 |  |
|  | by sex | Male | Female | Male | Female | Male | Female |
|  |  | *p* = 0.3775 | *p* = 0.7456 | *p* = 0.2306 | *p* = 0.1002 | *p* = 0.9885 | *p* = 0.3766 |
|  |  | r = 0.2803 | r = 0.0998 | r = 0.5769 | r = 0.5820 | r = 0.0067 | r = 0.3360 |
| Aβ(1-42) |  | *p* = 0.2489 |  | *p* = 0.7015 |  | *p* = 0.9378 |  |
|  |  | r = 0.2395 |  | r = 0.1080 |  | r = 0.0212 |  |
|  | by sex | Male | Female | Male | Female | Male | Female |
|  |  | *p* = 0.6122 | *p* = 0.2648 | *p* = 0.7879 | *p* = 0.9292 | *p* = 0.5979 | *p* = 0.8527 |
|  |  | r = 0.1632 | r = 0.3500 | r = 0.1424 | r = 0.0348 | r = 0.2441 | r = 0.0726 |
| Aβ(1-42)/Aβ(1-40) |  | *p* = 0.7284 |  | *p* = 0.7308 |  | ***p*** **= 0.0150** |  |
|  |  | r = 0.0731 |  | r = 0.0970 |  | **r = 0.5951** |  |
|  | by sex | Male | Female | Male | Female | Male | Female |
|  |  | *p* = 0.6914 | *p* = 0.9456 | *p* = 0.6643 | *p* = 0.5888 | *p* = 0.4463 | ***p* = 0.0083** |
|  |  | r = 0.1281 | r = 0.0211 | r = 0.2277 | r = 0.2093 | r = 0.3466 | **r = 0.8090** |
| Aβ(1-38+1-40)/Aβ(1-42) |  | *p* = 0.5094 |  | *p* = 0.9422 |  | *p* = 0.7232 |  |
|  |  | r = 0.1384 |  | r = 0.0205 |  | r = 0.0961 |  |
|  | by sex | Male | Female | Male | Female | Male | Female |
|  |  | *p* = 0.4739 | *p* = 0.5153 | *p* = 0.1924 | *p* = 0.7906 | *p* = 0.6254 | *p* = 0.8472 |
|  |  | r = 0.2290 | r = 0.1986 | r = 0.6165 | r = 0.0997 | r = 0.2265 | r = 0.0754 |

*Note: β-amyloid peptide = Aβ; early-onset Alzheimer disease = EOAD; late-onset Alzheimer disease = LOAD*
